# Knowledge, attitude, and practice toward fall risk-increasing drugs among nurses: a cross-sectional survey

**DOI:** 10.3389/fpubh.2025.1714722

**Published:** 2026-01-14

**Authors:** Minfang Zhu, Xiuli Ou, Xi’e Shu, Cuimin Lu, Meili Li, Lichang Gao, Caiying Zhao, Juanbi Gao

**Affiliations:** 1 Department of Neurology, Jiangmen Central Hospital, Jiangmen, China; 2 Department of Nursing, Jiangmen Central Hospital, Jiangmen, China; 3 Labour Union, Jiangmen Central Hospital, Jiangmen, China; 4 Elderly Care Service Team, Jiangmen Central Hospital, Jiangmen, China

**Keywords:** attitude, fall, fall risk-increasing drugs, knowledge, nurse, practice

## Abstract

**Introduction:**

Fall risk-increasing drugs (FRIDs) are a main risk factor for falls and fall-related injuries, particularly among inpatients. Nurses play a critical role in reducing fall risks and managing FRIDs within healthcare settings. In this study, we aimed to assess nurses’ knowledge, attitude, and practice (KAP) regarding FRIDs and identify the influencing factors.

**Methods:**

Based on the KAP theoretical framework, we developed a structured questionnaire to evaluate FRID-related KAP among nurses. An online survey was conducted using this questionnaire across 31 hospitals in Guangdong Province, China, between December 2024 and February 2025. Statistical analyses were performed using IBM SPSS 26.0. Univariate analysis and ordinal logistic regression analysis with logit as the link function were used for identifying influencing factors. Spearman correlation analysis was used for assessing relationships among knowledge, attitude, and practice.

**Results:**

Of the 600 nurses invited to participate in this survey, 542 (90.3%) completed the valid questionnaires. The median scores of nurses’ knowledge, attitude, and practice toward FRIDs were 56.0 [interquartile range (IQR): 47.0–67.0], 44.0 (IQR: 36.0–45.0) and 47.0 (IQR: 42.0–55.0), respectively. The training experience, department, and professional title were significant factors for nurses’ knowledge. Attitude was positively influenced by the training experience and professional title. Practice was mainly influenced by the training experience. Nurses’ knowledge was significantly associated with attitude (*r* = 0.476, *p* < 0.001) and practice (*r* = 0.551, *p* < 0.001), while their attitude was significantly associated with practice (*r* = 0.526, *p* < 0.001).

**Conclusion:**

Nurses demonstrated a moderate level of knowledge, positive attitude, and appropriate practices toward FRIDs. Training experience emerged as a consistent positive influencing factor across all three domains. Therefore, enhancing FRID-related training programs, through improved content design and incentive mechanisms, might effectively strengthen nurses’ KAP and contribute to better fall-prevention outcomes.

## 1 Introduction

Falls are among the most prevalent adverse events in inpatients (1). Recent evidence has shown that the rate of in-hospital falls is as high as 3.4–3.9%, posing a significant burden on both affected patients and the healthcare system (2–4). Multiple risk factors contribute to in-hospital falls, including both modifiable and non-modifiable factors. Among the modifiable factors, the use of fall risk-increasing drugs (FRIDs) is recognized as a major contributor.

FRIDs comprise a range of medications that negatively impact balance. These drugs are typically classified into three categories: cardiovascular drugs, psychotropics, and other drugs. Studies have identified that certain cardiovascular drugs, such as vasodilators, diuretics (including loop diuretics), calcium channel blockers, and alpha-adrenoceptor antagonists, are significantly associated with an increased fall risk (5). Among psychotropics, antipsychotics, antidepressants, and benzodiazepines are consistently associated with a higher fall risk (6). Other drugs, such as analgesics, nonsteroidal anti-inflammatory drugs, opioids, anti-Parkinson drugs, and antiepileptics, and polypharmacy are also significantly related to an increased fall risk (7).

Despite their known risks, FRIDs are widely used among inpatients. A retrospective study found that 83.2% of inpatients who experienced falls were prescribed FRIDs; moreover, patients using more than two FRIDs were 1.12 times more likely to fall compared with those not taking high-risk medications (8). The mechanism behind this risk is primarily related to the impact of these drugs on the cardiovascular or central nervous system, causing side effects such as orthostatic hypotension, bradycardia, sedation, sleep disturbances, confusion, and dizziness (9). Due to their clinical necessity, many FRIDs are prescribed during hospitalization and cannot be temporarily discontinued. Therefore, hospital nurses play a key role in managing FRIDs to recognize and prevent their side effects.

The knowledge, attitude, and practice (KAP) theory is a theoretical framework widely used in healthcare research that comprises three interconnected stages: Acquiring knowledge, developing attitude, and forming behaviors (10). The theory positions behavior change as the ultimate goal, with knowledge as the foundation, and attitude as the primary driving forces that lead to behavioral transformation (11). Several studies have applied the KAP framework to investigate nurses’ roles in medication management (12–14). However, few studies have specifically explored nurses’ management of FRIDs using the KAP theory. In clinical practice, nurses play a crucial role in reducing in-hospital fall risks and managing FRIDs. Referring to previous literature on drug management (14), nurses should be aware of FRID-related nursing responsibilities. By using the KAP theory, the connotation of each dimension can be clearly delineated and the FRID-related nursing responsibilities can be determined. The knowledge component refers to nurses’ understanding of the types and quantities of FRIDs, as well as their awareness of the potential fall-related risks. The attitude component involves nurses’ perspective on proactively mastering the indication, types, and adverse effects of FRIDs, implementing interventions. The practice component encompasses nurses’ practical actions in response to FRID-related risks, such as conducting medication risk assessments, implementing timely fall-prevention measures, and delivering specific actions for targeted health education. This could help nurses clearly grasp the corresponding nursing responsibilities based on the content of each dimension.

In this study, we developed a questionnaire based on the KAP theory and conducted an online survey using this questionnaire. The aims of this study were to explore the knowledge, attitude, and practice regarding FRIDs among nurses and determine the factors influencing these domains. This could help strengthen FRID management and provide theoretical evidence for reducing FRID-related falls.

## 2 Methods

### 2.1 Study design

A descriptive cross-sectional study was conducted with an online survey. The study was reported following the Strengthening the Reporting of Observational studies in Epidemiology (STROBE) guideline. This study was reviewed and approved by the Ethics Committee of Jiangmen Central Hospital (No. 2024159).

### 2.2 Study setting and participants

This study was conducted in 31 hospitals in Guangdong Province, China, between December 2024 and February 2025. Participants were recruited through convenience sampling. The inclusion criteria were: ≥18 years of age, possession of a valid Chinese nurse practitioner certificate, had ≥1 year clinical nursing experience, worked in hospitals in China as a nurse, and willingness to participate voluntarily. The exclusion criteria were: Nurses who had left clinical practice for >1 year and those who withdrew from the survey.

### 2.3 Questionnaire development

Based on the KAP framework, we created an initial pool of 42 items associated with FRIDs. Content validation was conducted using the Delphi technique. Invitations and descriptions about the content were emailed to experts who had agreed to participate voluntarily. A total of eight experts from six different hospitals, each with 15–41 years of clinical nursing experience, participated in the expert consultations. In each round, experts independently reviewed the content and scored each item using a 5-point Likert scale (1 = not important, 5 = very important). After two rounds of expert consultations, eight items were removed due to a total score of <80%, resulting in a final questionnaire with 34 items.

The questionnaire was presented in Chinese and comprised four parts: (1) demographic characteristics of the participants, which included nine questions; (2) knowledge about FRIDs, which consisted of 14 questions rated on a 5-point Likert scale (1 = very unclear and 5 = very clear), with a total score ranging between 14 and 70 points; (3) attitude regarding FRIDs, which consisted of nine questions rated on a 5-point Likert scale (1 = strongly disagree and 5 = strongly agree), with a total score ranging between 9 and 45 points; (4) practice related to FRIDs, which consisted of 11 questions rated on a 5-point Likert scale (1 = never and 5 = always), with a total score ranging between 11 and 55 points. The total possible score ranged between 34 and 170 points, with higher scores indicating a greater level of knowledge, more positive attitude, and more rigorous practice.

In this study, scores were divided based on the principle of one-third of each degree. Knowledge scores were divided into three degrees, including a low knowledge level (14–32 points), medium knowledge level (33–51 points), and high knowledge level (52–70 points). Scores for attitude were separated into three degrees, comprising a low (9–20 points), medium (21–32 points), and high (33–45 points) attitude level, whereas scores for practice were divided into three degrees, consisting of a low (11–25 points), medium (26–40 points), and high (41–55 points) practice level. To evaluate the reliability and refine the wording, a pilot test was conducted with 30 randomly selected nurses who met the inclusion and exclusion criteria in Jiangmen Central hospital. Based on their feedback, the phrasing of two items was revised. The final questionnaire had a Cronbach’s *α* of 0.950. The total content validity was 0.952; content validity of each dimension ranged between 0.931 and 0.966.

### 2.4 Data collection

The questionnaire was distributed using the online platform Questionnaire Star. Through this platform, a QR code of the questionnaire was generated. The participants who accepted the survey invitation received the QR code through WeChat. By scanning the QR code, the participants were directed to a page outlining the purpose and significance of the study, after which they proceeded to complete the questionnaire following the prompts. To ensure data quality, each questionnaire could only be completed once per IP address, and all items had to be filled out before submission. The completed data were exported in Excel format from the Questionnaire Star platform.

Data collection was conducted in 31 hospitals of varying grades across Guangdong Province. Based on a previous investigation (15), hospitals were selected using convenience sampling; furthermore, survey invitations were distributed to approximately 5% of the total nursing staff at each institution. As a result, 600 nurses were invited to participate. Completing the questionnaire required approximately 8–10 min. If the participant provided inconsistent information, the questionnaire was considered invalid.

### 2.5 Statistical analyses

Statistical analyses were performed using IBM SPSS 26.0 software (IBM, Armonk, NY, United States). Demographic characteristics are summarized using frequencies, means, standard deviations and percentages. Scores of knowledge, attitude, and practice are described using medians and interquartile ranges (IQRs). The relationships among knowledge, attitude, and practice scores were assessed by Spearman correlation analysis. The Mann–Whitney U test or Kruskal–Wallis H test was used for evaluating the influence of each characteristic on knowledge, attitude, and practice scores. Variables identified as significant using univariate analysis were fitted in the ordinal logistic regression. We developed three ordinal logistic regression models: one for knowledge, one for attitude, and one for practice. Dependent variables included the knowledge, attitude, and practice score levels, which were all presented as a low, medium, and high knowledge level. The multicollinearity of independent variables was completed in the early stages of modeling, and all of the models were developed using logit as the link function. If the tolerance was less than 0.1 or the variance inflation factor (VIF) was greater than 10, the presence of collinearity was indicated. All statistical tests were two-sided, and a *p* < 0.05 was considered statistically significant in all analyses.

## 3 Results

### 3.1 Demographic characteristics of participants

In total, 600 nurses from 31 medical institutions of different grades (primary, secondary, and tertiary) in Guangdong Province were invited to participate in this survey. Of these, 563 (93.8%) nurses responded. In total, 542 (90.3%) valid questionnaires were included in the final analysis, while 21 invalid questionnaires were excluded because of inconsistent information. Table 1 presents the demographic characteristics of these 542 participants. The majority of participants (97.8%) were female nurses. The mean age was 32.93 ± 8.35 years; the mean duration of nursing experience was 12.00 ± 8.71 years. Most participants (75.1%) had received training related to FRIDs.

**Table 1:** Demographic characteristics of participants (*N* = 542).

Variables	*N*	%
Sex		
Male	11	2.2
Female	531	97.8
Age, years		
18–30	266	49.1
31–40	172	31.7
41–50	92	17.0
>50	12	2.2
Education level		
Junior college and below	243	44.8
Bachelor degree and above	299	55.2
Years of nursing experience, years		
<5	103	19.0
5–9	164	30.3
10–14	97	17.9
≥15	178	32.8
Professional title		
Nurse	155	28.6
Nurse practitioner	199	36.7
Nurse in charge	146	26.9
Associate chief nurse and above	42	7.8
Hospital type		
General hospital	399	73.6
Speciality hospital	32	5.9
Other	111	20.5
Hospital grade		
Class III	225	41.5
Class II	218	40.2
Class I	99	18.3
Department		
Internal medicine department	280	51.7
Surgical department	132	24.4
Women and children’s department	44	8.1
Other	86	15.8
Have you received fall risk increasing drug knowledge training		
Yes	407	75.1
No	135	24.9

### 3.2 Nurses’ knowledge of FRIDs

Details on the items, levels, and scores related to FRID knowledge are presented in Table 2. The median score of nurses’ knowledge about FRIDs was 56.0 (IQR: 47.0–67.0) points, indicating a moderate level of knowledge. As many as 39 (7.2%) nurses reported limited knowledge about the types of FRIDs, and 21(3.9%) nurses reported limited knowledge about the negative impact of FRIDs. Compared with other FRID categories, nurses demonstrated lower knowledge about specific medications, including antidepressants, antihypertensive medications, and medications for treating bladder overactivity, urinary incontinence, and related conditions.

**Table 2:** Nurses’ knowledge of fall risk increasing drugs (*N* = 542).

Items	Very unclear N(%)	Unclear N(%)	Neutral N(%)	Clear N(%)	Very clear N(%)	Score	
						Median	IQR (Q1, Q3)
1. Do you know the types of fall risk-increasing drugs?	7(1.3)	32(5.9)	182 (33.6)	207 (38.2)	114 (21.0)	4	1 (3, 4)
2. Do you know that fall risk-increasing drugs increase the risk of falling?	5 (0.9)	16 (3.0)	144 (26.6)	184 (33.9)	193 (35.6)	4	2 (3, 5)
3. Do you know that antipsychotic medications (such as olanzapine and quetiapine) can increase the risk of falls?	7(1.3)	29 (5.3)	124 (22.9)	181 (33.4)	201 (37.1)	4	2 (3, 5)
4. Do you know that antidepressants (such as sertraline and amitriptyline) can increase the risk of falls?	5 (0.9)	36 (6.6)	143 (26.4)	181 (33.4)	177 (32.7)	4	2 (3, 5)
5. Do you know that antiepileptic medications (such as carbamazepine and sodium valproate) can increase the risk of falls?	3 (0.6)	27 (5.0)	138 (25.5)	177 (32.6)	197 (36.3)	4	2 (3, 5)
6. Do you know that sedatives (such as escitalopram and diazepam) can increase the risk of falls?	3 (0.6)	8 (1.5)	106 (19.5)	170 (31.4)	255 (47.0)	4	1 (4, 5)
7. Do you know that loop diuretics (such as furosemide and torasemide) can increase the risk of falls?	3 (0.6)	26 (4.8)	116 (21.4)	174 (32.1)	223 (41.1)	4	2 (3, 5)
8. Do you know that anesthetics (such as fentanyl and pethidine) can increase the risk of falls?	4 (0.8)	12 (2.2)	109 (20.1)	174 (32.1)	243 (44.8)	4	1 (4, 5)
9. Do you know that α blockers (such as phentolamine and prazosin) can the risk of falls?	5 (0.9)	37 (6.9)	143 (26.4)	166 (30.6)	191 (35.2)	4	2 (3, 5)
10. Do you know that vasodilators (such as nitroglycerin and sodium nitroprusside) can increase the risk of falls?	5 (0.9)	13 (2.4)	119 (22.0)	181 (33.4)	224 (41.3)	4	2 (3, 5)
11. Do you know that central antihypertensive medications (such as clonidine and methyldopa) can increase the risk of falls?	6 (1.1)	41 (7.6)	138 (25.4)	175 (32.3)	182 (33.6)	4	2 (3, 5)
12. Do you know that antihistamines (such as diphenhydramine and promethazine) can increase the risk of falls?	4 (0.8)	30 (5.5)	121 (22.3)	183 (33.8)	204 (37.6)	4	2 (3, 5)
13. Do you know that medications for treating bladder overactivity, urinary incontinence, and related conditions (such as oxybutynin and Mirabillon) can increase the risk of falls?	9 (1.7)	51 (9.4)	158 (29.1)	174 (32.1)	150 (27.7)	4	2 (3, 5)
14. Do you know that the more fall risk-increasing drugs used simultaneously, the higher the risk of falls?	3 (0.6)	13 (2.4)	112 (20.6)	174 (32.1)	240 (44.3)	4	1 (4, 5)

IQR, interquartile range.

### 3.3 Nurses’ attitude toward FRIDs

Table 3 summarizes the agreement levels and scores for nurses’ attitudes. The median score for nurses’ attitude toward FRIDs was 44.0 (IQR: 36.0–45.0) points, indicating that nurses have a good attitude toward FRIDs. Most nurses (>60%) strongly agreed with the necessity for learning about FRIDs, mastering the types of medications, being familiar with their adverse reactions, and providing fall prevention education to patient prescribed with FRIDs.

**Table 3:** Nurses’ attitude toward fall risk increasing drugs (*N* = 542).

Items	Very disagree N(%)	Disagree N(%)	Neutral N(%)	Agree N(%)	Very agree N(%)	Score	
						Median	IQR (Q1, Q3)
1. Do you think it is necessary for nurses to learn about fall risk-increasing drugs?	1 (0.2)	0 (0.0)	39 (7.2)	146 (26.9)	356 (65.7)	5	1 (4, 5)
2. Do you think it is necessary for nurses to master the types of fall risk-increasing drugs?	1 (0.2)	1 (0.2)	42 (7.7)	155 (28.6)	343 (63.3)	5	1 (4, 5)
3. Do you think it is necessary for nurses to be familiar with the adverse reactions of fall risk-increasing drugs?	1 (0.2)	0 (0.0)	42 (7.7)	163 (30.1)	336 (62.0)	5	1 (4, 5)
4. Do you think it is necessary to regularly evaluate whether the medication used by the patient is a fall risk-increasing drug?	2 (0.4)	1 (0.2)	50 (9.2)	169 (31.2)	320 (59.0)	5	1 (4, 5)
5. Do you think it is necessary to regularly evaluate the fall risk of patients using fall risk-increasing drugs?	1 (0.2)	0 (0.0)	53 (9.8)	173 (31.9)	315 (58.1)	5	1 (4, 5)
6. Do you think it is necessary to provide fall prevention education to patients using fall risk-increasing drugs?	1 (0.2)	0 (0.0)	41 (7.6)	165 (30.4)	335 (61.8)	5	1 (4, 5)
7. Do you think it is necessary to restrict the activity of a patient using fall risk-increasing drugs? (For example, suggesting bed rest, using wheelchairs or lathes for external checkups, etc.)?	2 (0.4)	2 (0.4)	54 (10.0)	172 (31.7)	312 (57.5)	5	1 (4, 5)
8. Do you think it is necessary to analyze the impact of fall risk-increasing drugs on falls?	2 (0.4)	1 (0.2)	54 (10.0)	171 (31.5)	314 (57.9)	5	1 (4, 5)
9. Do you think that paying attention to the use of fall risk-increasing drugs can reduce the risk of falls?	1 (0.2)	3 (0.6)	56 (10.3)	168 (31.0)	314 (57.9)	5	1 (4, 5)

IQR, interquartile range.

### 3.4 Nurses’ practice toward FRIDs

The findings on nurses’ practices are presented in Table 4. The median score for nurses’ practice toward FRIDs was 47.0 (IQR: 42.0–55.0) points, suggesting an appropriate level of practice. More than half of the participants reported that they consistently performed several key behaviors, including providing patients with fall prevention measures, providing a wheelchair or lathe for patients when going out for examinations after FRID administration, and advising patients to rise slowly, discontinuing FRID treatment.

**Table 4:** Nurses’ practice toward fall risk increasing drugs (*N* = 542).

Items	Never N(%)	Rarely N(%)	Sometimes N(%)	Often N(%)	Always N(%)	Score	
						Median	IQR (Q1, Q3)
1. Would you evaluate whether the medication used by the patient is a fall risk-increasing drug?	6 (1.1)	13 (2.4)	110 (20.3)	195 (36.0)	218 (40.2)	4	1 (4, 5)
2. Would you assess the number of fall risk-increasing drugs received by the patient simultaneously?	7(1.3)	22 (4.1)	113 (20.8)	202 (37.3)	198 (36.5)	4	2 (3, 5)
3. Would you reassess the patients’ risk of falls when they are receiving fall risk-increasing drugs?	10 (1.8)	20 (3.7)	95 (17.6)	185 (34.1)	232 (42.8)	4	1 (4, 5)
4. Would you promptly provide fall prevention education to patients receiving fall risk-increasing drugs?	5 (0.9)	10 (1.9)	74 (13.6)	201 (37.1)	252 (46.5)	4	1 (4, 5)
5. Would you promptly remind patients to rest in bed when they are using fall risk-increasing drugs in the ward?	3 (0.6)	8 (1.5)	65 (12.0)	197 (36.3)	269 (49.6)	4	1 (4, 5)
6. Would you promptly provide patients with fall prevention measures (such as using the bedrail during bed rest) when they are using fall risk-increasing drugs in the ward?	3 (0.6)	4 (0.7)	58 (10.7)	185 (34.1)	292 (53.9)	5	1 (4, 5)
7. Would you provide a wheelchair or lathe for patients to go out for examination after using fall risk-increasing drugs?	3 (0.6)	7(1.3)	58 (10.7)	182 (33.5)	292 (53.9)	5	1 (4, 5)
8. Would you reassess the patients’ fall risk when they have just discontinued treatment with fall risk-increasing drugs?	12 (2.2)	15 (2.8)	79 (14.6)	185 (34.1)	251 (46.3)	4	1 (4, 5)
9. Would you guide patients to wake up slowly when they have just discontinued treatment with fall risk-increasing drugs?	5 (0.9)	4 (0.7)	70 (12.9)	181 (33.4)	282 (52.1)	5	1 (4, 5)
10. Do you proactively consider the impact of fall risk-increasing drugs when a fall occurs?	7(1.3)	9 (1.7)	99 (18.2)	206 (38.0)	221 (40.8)	4	1 (4, 5)
11. Would you analyze the effects of fall risk-increasing drugs when reporting adverse events of falls?	5 (0.9)	15 (2.8)	90 (16.6)	195 (36.0)	237 (43.7)	4	1 (4, 5)

IQR, interquartile range.

### 3.5 Correlation analysis

Correlation analysis revealed that knowledge was significantly associated with both attitude (*r* = 0.476, *p* < 0.001) and practice (*r* = 0.551, *p* < 0.001). Additionally, attitude was significantly associated with practice (*r* = 0.526, *p* < 0.001).

### 3.6 Univariate analysis

Table 5 presents the results of the univariate analysis of factors associated with knowledge, attitude, and practice. Nurses’ knowledge was significantly associated with six factors, including education level (*p* < 0.001), professional title (*p* < 0.001), hospital type (*p* < 0.001), hospital grade (*p* < 0.001), department (*p* = 0.021), and relevant training experience (*p* < 0.001). Nurses’ attitudes were significantly associated with six factors, including sex (*p* = 0.023), education level (*p* = 0.002), professional title (*p* = 0.016), hospital type (*p* = 0.019), hospital grade (*p* = 0.006), and relevant training experience (*p* < 0.001). Nurses’ practice was significantly related to nursing experience (*p* = 0.046), hospital grade (*p* = 0.004), and relevant training experience (*p* < 0.001).

**Table 5:** Univariate analysis of nurses’ knowledge, attitude, and practice of fall risk increasing drugs (*N* = 542).

Variables	Knowledge		Attitude		Practice	
	Mean rank	P*	Mean rank	P*	Mean rank	P*
Sex		0.296		**0.023**		0.084
Male	222.86		172.64		192.05	
Female	272.51		273.55		273.15	
Age, years		0.078		0.451		0.220
18–30	254.16		265.36		260.65	
31–40	288.76		272.77		277.24	
41–50	284.03		279.34		383.30	
>50	312.38		329.33		339.29	
Education level		<**0.001**		**0.002**		0.190
Junior college and below	238.45		250.34		261.87	
Bachelor degree and above	298.36		288.70		279.33	
Years of nursing experience, years		0.056		0.284		**0.046**
<5	252.61		264.87		269.57	
5–9	269.54		269.02		261.95	
10–14	250.51		253.71		244.87	
≥15	295.68		287.31		295.93	
Professional title		<**0.001**		**0.016**		0.397
Nurse	248.18		253.66		262.23	
Nurse practitioner	254.01		274.34		265.99	
Nurse in charge	303.03		268.48		279.88	
Associate chief nurse and above	330.82		334.36		302.70	
Hospital type		<**0.001**		**0.019**		0.065
General hospital	288.57		280.98		279.43	
Speciality hospital	287.67		273.39		278.30	
Other	205.50		236.89		241.03	
Hospital grade		<**0.001**		**0.006**		**0.004**
Class III	297.38		283.95		270.86	
Class II	281.67		277.66		291.27	
Class I	190.30		229.64		229.42	
Department		**0.021**		0.069		0.207
Internal medicine department	281.74		281.70		275.21	
Surgical department	280.67		265.63		273.28	
Women and children’s department	272.14		290.98		298.82	
Other	223.76		237.34		242.72	
Have you received fall risk increasing drug knowledge training		<**0.001**		<**0.001**		<**0.001**
Yes	306.77		288.37		301.58	
No	165.17		220.65		180.82	

*P < 0.05 was considered statistically significant. Bold values introduction to *P*-values.

### 3.7 Ordinal logistic regression analysis

The significant independent factors were further analyzed using ordinal logistic regression analysis. In the multicollinearity evaluation, the tolerance of independent variables ranged between 0.112 and 0.948; the VIFs ranged between 1.059 and 8.913 in the knowledge model. Similarly, the tolerance of independent variables ranged between 0.112 and 0.958 and the VIFs ranged between 1.043 and 8.913 in the attitude model. The tolerance of independent variables ranged between 0.847 and 0.961 and the VIFs ranged between 1.041 and 1.224 in the practice model. All tolerance values were greater than 0.1; moreover, all the VIF values were less than 10; therefore, there was no collinearity.

The positive factors are presented in Table 6. In the knowledge model, the −2 log likelihood value was 153.791(*p <* 0.001), and the goodness-of-fit test was satisfactory (Pearson, *p* = 0.454; deviance, *p* = 0.999). The professional title, department, and training experience were positive factors for nurses’ knowledge. Compared with those of associate chief nurses and above, nurses [odds ratio (OR) = 0.182, *p* = 0.004] and nurse practitioners (OR = 0.217, *p* = 0.007) had significantly lower knowledge scores. Participants in the internal medicine department (OR = 2.186, *p* = 0.012) had higher knowledge scores than did those in other departments. Participants who had training experience (OR = 9.806, *p* < 0.001) had higher knowledge scores than did those who had not received training. In the attitude model, the −2 log likelihood value was 41.943 (*p* < 0.001), and the goodness-of-fit test was good (Pearson, *p* = 1.000; deviance, *p* = 1.000). The positive factors for nurses’ attitudes were the professional title and training experience. Using associate chief nurses and above as the reference, nurses, nurse practitioners, and nurses in charge had relatively lower attitude levels because their OR values were all less than 0.001. Nurses with training experience (OR = 4.367, *p* < 0.001) had a better attitude toward FRIDs. In the practice model, the −2 log likelihood value was 55.767 (*p* < 0.001), and the goodness-of-fit test was good (Pearson, *p* = 0.709; deviance, *p* = 0.467). Training experience was also a positive factor for practice. Nurses who had received FRID training experience (OR = 5.099, *p <* 0.001) had a better practice level than did those without FRID training experience.

**Table 6:** Ordinal logistic regression analysis of nurses’ knowledge, attitude, and practice of fall related medications (*N* = 542).

Variables	OR	Estimate	95%CI	P*
Knowledge				
Professional title (nurse)	0.182	−1.703	−2.866 ~ −0.540	**0.004**
Professional title (nurse practitioner)	0.217	−1.530	−2.652 ~ −0.409	**0.007**
Professional title (nurse in charge)	0.349	−1.053	−2.184 ~ 0.079	0.068
Professional title (associate chief nurse and above)	Reference			
Department (internal medicine department)	2.186	0.782	0.173 ~ 1.391	**0.012**
Department (surgical department)	1.550	0.438	−0.247 ~ 1.123	0.210
Department (women and children’s department)	1.405	0.340	−0.516 ~ 1.195	0.437
Department (other)	Reference			
Have you received fall related medication knowledge training				
Yes	9.806	2.283	1.816 ~ 2.749	<**0.001**
No	Reference			
Attitude				
Professional title (nurse)	1.747 × 10^−8^	−17.863	−18.772 ~ −16.954	<**0.001**
Professional title (nurse practitioner)	2.358 × 10^−8^	−17.563	−18.435 ~ −16.692	<**0.001**
Professional title (nurse in charge)	2.353 × 10^−8^	−17.565	−18.431 ~ −16.688	<**0.001**
Professional title (associate chief nurse and above)	Reference			
Have you received fall related medication knowledge training				
Yes	4.367	1.474	0.830 ~ 2.118	<**0.001**
No	Reference			
Practice				
Have you received fall related medication knowledge training				
Yes	5.099	1.629	1.163 ~ 2.095	<**0.001**
No	Reference			

*P < 0.05 was considered statistically significant. Bold values introduction to *P*-values.

## 4 Discussion

Although previous studies have reported the KAP regarding falls among medical staff, many key aspects of FRIDs remain under-addressed. In this study, we focused on the KAP regarding FRIDs among nurses from 31 medical institutions of varying grades in China, providing new insights and perspectives. Our findings revealed that nurses had a moderate level of knowledge, positive attitudes, and appropriate practice regarding FRIDs.

Our study focused on promoting and refining the management of FRIDs among nurses. Although there have been studies on fall prevention or medication safety in the past (13, 16), these studies have not addressed the refined management of FRIDs among nurses. For instance, in terms of knowledge, the mechanisms of various common FRIDs were included, and the results could suggest which drugs with relatively low scores we should consider more attention. However, adverse reactions and regular evaluations of FRIDs were included in the attitude and practice part. These detailed perspectives could contribute to the implementation of FRID management.

This study showed that nurses had a moderate level of knowledge about FRIDs but poor knowledge about the types of FRIDs. Specifically, nurses demonstrated lower scores for items associated with drugs that required long-term use, including antidepressants, antihypertensive medications, and medications for treating bladder overactivity, urinary incontinence, and related conditions. Although recent studies suggested the potential for discontinuing FRIDs, such interventions could not be implemented arbitrarily or within a short period. For instance, a prospective cohort study showed that it took at least 2 months to discontinue or reduce the dosage of FRIDs for an individual patient, with a minimal impact on the fall risk (17). A similar finding was reported from a randomized controlled trial conducted in four European countries (18). In another controlled trial with a 1-year follow-up period, only a small proportion of patients who received medication consultation were able to discontinue high-risk medications or reduce their dosage (19). Given the challenges associated with discontinuation and their clinical necessity, it is important for nurses to enhance their knowledge and understanding of all types of FRIDs to ensure safe and effective nursing care.

Our logistic regression analysis revealed that receiving training, the department type, and the professional title were significant factors for knowledge about FRIDs. Receiving training, either actively or passively, is a primary method for nurses to acquire and improve knowledge. Systemic and repetitive training enhances the knowledge and effectiveness of a training program, which was emphasized in a qualitative study (20). Regarding department types, we found that nurses who worked in departments of internal medicine had higher scores than did those working in other departments. This finding is consistent with findings from a cross-sectional study in Saudi Arabia, and this may be related to the health status of patients treated in the internal medicine department (21). Patients from general wards are more mobile and less likely to be bedridden for prolonged periods, despite not having fully recovered. This increases their risk of in-hospital falls. To avoid fall events, nurses working in these departments may be more proactive in acquiring knowledge about FRIDs. Additionally, nurses with a higher professional title had higher knowledge about FRIDs. This might be because most nurses with higher professional titles had more pharmacological and nursing experience. In addition, the length of experience may influence knowledge.

Although nurses’ knowledge about FRIDs was found to be moderate, most nurses demonstrated a positive attitude toward FRIDs. The highest score was given to the item “Do you think it is necessary for nurses to learn about FRID knowledge,” indicating a consistent willingness of nurses to actively acquire knowledge about FRIDs. A cross-sectional survey in China reported unexpected findings that nurses scored the lowest on the item “Falls are preventable” (22). However, 88.9% of participants in our study expressed agreement with the viewpoint that the fall incidence could be reduced by paying attention to the use of FRIDs. In addition, nurses in our study also suggested that regular evaluations and timely education regarding FRIDs were necessary for preventing falls. This discrepancy may be due to differences in the way questions were phrased. When asked about whether falls could be prevented, nurses may consider the complexity of multiple factors, leading to a negative attitude. In contrast, when asked about a specific preventive factor, for example, the use of FRIDs, nurses expressed a more realistic and supportive attitude.

Our analysis revealed that training experience and higher professional titles were associated with more positive attitudes toward FRIDs. Nurses’ training experience, which includes receiving educational interventions, appears to be particularly influential. An observational study conducted in India reported that educational interventions increased nurses’ attitude toward pharmacovigilance from 72.86 to 85.92% (23), revealing the positive effect of training experience on attitude. Regarding the professional title, previous studied proposed that nurses holding senior professional titles had higher scores for attitude toward FRIDs (16, 24). In our study, the senior professional title was a positive factor for nurses’ attitudes toward FRIDs, but the OR values between groups were very small. This may be because the attitude scores of the participants in this study tended to be at a higher level.

Our study found that nurses demonstrated appropriate practice regarding FRIDs. A case–control study previously confirmed that the number of FRIDs is one of the most important predictors of the inpatient fall risk (25). Compared with the timely implementation of fall prevention measures and targeted health education, nurses in our study scored lowest on items related to FRID risk assessments, particularly on items related to assessments of the number of FRIDs. This may be explained by several reasons. First, most nurses were not familiar with all types of FRIDs and tended to be familiar only with the commonly used FRIDs in their own departments. Second, limited opportunities were offered for nurses to proactively learn about FRIDs. Additionally, insufficient attention from nursing managers toward FRID risk management may also contribute to this observation.

Logistic regression analysis revealed that receiving training was a significant factor influencing nurses’ practice toward FRIDs. To promote the quality of practice, previous studies have proposed the innovation and reform of nurse training methods. One study showed that well-structured training equips nurses with the necessary skills to manage patients at a high risk of falls, including those administered FRIDs (26). However, nurse training should place greater emphasis on content reform, both in terms of improving the educational materials and aligning them with the nursing scope of practice (27). Another study suggested that developing educational training through the academic curriculum and continuing educational programs could improve nurses’ practice (28). The aforementioned measures to reform training on FRIDs may enhance nurses’ practical ability regarding FRIDs.

Regarding the impact of knowledge on practice, prior research has proposed that a high knowledge level could improve practice (29). Actually, accessing more information about FRIDs can improve nurses’ knowledge, which may subsequently improve their practice. Further, a more positive attitude could also promote practice in principle. Our correlation analysis revealed moderate correlations among nurses’ knowledge, attitude, and practice toward FRIDs. This may be because the practical rate of knowledge transformation is not always speedy. Participants are aware of the importance of FRIDs but do not have a deep understanding of the specific principles, resulting in limited translation into practice. The second reason was because of the constraints of the practical environment. Though many participants have positive attitudes toward FRIDs, a lack of institutional guidance of FRID use and insufficient leadership support for FRID management might affect the rate of conversion from attitude to practice.

This study had some limitations. First, we evaluated the questionnaire and found that it had good Cronbach’s *α* and content validity. However, we did not use factor analysis to further understand the structural validity of the questionnaire. Second, the participants were recruited through convenience sampling from 31 hospitals in Guangdong. Compared with random sampling or systematic sampling, convenience sampling is less rigorous for generalizability. Owing to limited team members in this study, we still adopted convenience sampling during the investigation. To compensate for the lack of convenient sampling, we have tried our best to include multiple hospitals of different grades and levels. Table 1 presents that 41.5% of the participants were from hospitals of Class III, 40.2% of participants were from hospitals of Class II and 18.3% of the participants were from hospitals of Class I, proving that the inclusion of participants was reasonable. The results could thus offer insight into the knowledge, attitudes, and practices of nurses across a representative range of institutions. Third, as a cross-sectional survey, the study did not involve any intervention measures and therefore could not assess changes over time. However, our findings provide a foundation for designing future interventions aimed at improving nurses’ knowledge, attitude, and practice toward FRIDs. Finally, the statistical results are limited to a single time point and cannot illustrate changes in individual nurse’s knowledge, attitude, and practice toward FRIDs over time. Further qualitative or longitudinal studies should be conducted to explore changes in characteristics and barriers of nurses’ knowledge, attitude, and practice toward FRIDs.

## 5 Conclusion

Although nurses demonstrated positive attitudes toward FRIDs, their knowledge was at a moderate level, and their practice was deemed appropriate. The influencing factors for nurses’ knowledge, attitude, and practice toward FRIDs were not consistent; however, participation in FRID-related training emerged as a common positive factor among the three domains. Additionally, while variables such as the professional title, hospital grade, department type, and education level are relatively objective, training is a relatively subjective and modifiable factor that can be addressed through intervention measures. Therefore, healthcare institutions should strengthen their support for training reform, particularly in terms of content design and the implementation of incentive mechanisms. Besides, there were limitations in sampling and region in this study, due to the convenience sampling method and the Guangdong province region. Future research could focus on these areas to improve nurses’ knowledge, attitude, and practice toward FRIDs.

## Data Availability

The raw data supporting the conclusions of this article will be made available by the authors, without undue reservation.

## References

[1] Solares NP Calero P Connelly CD. Patient perception of fall risk and fall risk screening scores. J Nurs Care Qual. (2023) 38:100–6. doi: 10.1097/NCQ.0000000000000645, 36094277

[2] Bernet NS Everink IHJ Hahn S Bauer S Schols JMGA. Comparing risk-adjusted inpatient fall rates internationally: validation of a risk-adjustment model using multicentre cross-sectional data from hospitals in Switzerland and Austria. BMC Health Serv Res. (2024) 24:331. doi: 10.1186/s12913-024-10839-x, 38481303 PMC10935870

[3] Davis J Casteel C Peek-Asa C. In-hospital fall and fracture risk with conditions in the elixhauser comorbidity index: an analysis of state inpatient data. J Patient Saf. (2021) 17:e1779–84. doi: 10.1097/PTS.0000000000000637, 32168270

[4] Dykes PC Curtin-Bowen M Lipsitz S Franz C Adelman J Adkison L . Cost of inpatient falls and cost-benefit analysis of implementation of an evidence-based fall prevention program. JAMA Health Forum. (2023) 4:e225125. doi: 10.1001/jamahealthforum.2022.5125, 36662505 PMC9860521

[5] de Vries M Seppala LJ Daams JG van de Glind EMM Masud T van der Velde N . Fall-risk-increasing drugs: a systematic review and meta-analysis: I. Cardiovascular drugs. J Am Med Dir Assoc. (2018) 19:371.e1–9. doi: 10.1016/j.jamda.2017.12.013 29396189

[6] Seppala LJ Wermelink AMAT de Vries M Ploegmakers KJ van de Glind EMM Daams JG . Fall-risk-increasing drugs: a systematic review and meta-analysis: ii. Psychotropics. J Am Med Dir Assoc. (2018) 19:371.e11–7. doi: 10.1016/j.jamda.2017.12.098 29402652

[7] Seppala LJ van de Glind EMM Daams JG Ploegmakers KJ de Vries M Wermelink AMAT . Fall-risk-increasing drugs: a systematic review and meta-analysis: iii. Others. J Am Med Dir Assoc. (2018) 19:372.e1–8. doi: 10.1016/j.jamda.2017.12.099 29402646

[8] Taylor B Tymkew H Vyers K Taylor M Roney W Costantinou E. Implementation of fall preventions over the past 15 years: impact on inpatient injury and insights for the future. J Nurs Care Qual. (2020) 35:365–71. doi: 10.1097/NCQ.0000000000000468, 31972784

[9] Lee J Negm A Peters R Wong EKC Holbrook A. Deprescribing fall-risk increasing drugs (FRIDs) for the prevention of falls and fall-related complications: a systematic review and meta-analysis. BMJ Open. (2021) 11:e035978. doi: 10.1136/bmjopen-2019-035978, 33568364 PMC7878138

[10] Zhou H Zhao R Yang Y. A qualitative study on knowledge, attitude, and practice of nursing students in the early stage of the Covid-19 epidemic and inspiration for nursing education in mainland China. Front Public Health. (2022) 10:845588. doi: 10.3389/fpubh.2022.845588, 35359792 PMC8964035

[11] Wang R Song Y He Y Long S Feng L. Status of knowledge, attitude and practice of poststroke dysphagia in neurological nurses in China: a cross-sectional study. PLoS One. (2023) 18:e0284657. doi: 10.1371/journal.pone.0284657, 37083919 PMC10121028

[12] Salehi T Seyedfatemi N Mirzaee MS Maleki M Mardani A. Nurses’ knowledge, attitudes, and practice in relation to pharmacovigilance and adverse drug reaction reporting: a systematic review. Biomed Res Int. (2021) 2021:6630404. doi: 10.1155/2021/6630404, 33937402 PMC8062168

[13] Giannetta N Dionisi S Cassar M Trapani J Renzi E Di Simone E . Measuring knowledge, attitudes and behavior of nurses in medication management: cross-cultural comparisons in Italy and Malta. Eur Rev Med Pharmacol Sci. (2020) 24:5167–75. doi: 10.26355/eurrev_202005_21212, 32432782

[14] Zhao W Xu Y Liu R Zhao T Ning Y Feng Y . Knowledge, attitudes, and practices of bedside nurses regarding antimicrobial stewardship in China: an explanatory sequential mixed methods study. J Nurs Manag. (2023) 2023:9059920. doi: 10.1155/2023/9059920, 40225635 PMC11919010

[15] Liu Y Zhang Z Liu Y. Knowledge, attitude, and practice toward the prevention of occupational exposure in public health emergencies among nurses in Wuhan. Front Public Health. (2024) 12:1289498. doi: 10.3389/fpubh.2024.1289498, 38645460 PMC11026623

[16] Cho MY Jang SJ. Nurses’ knowledge, attitude, and fall prevention practices at South Korean hospitals: a cross-sectional survey. BMC Nurs. (2020) 19:108. doi: 10.1186/s12912-020-00507-w, 33292192 PMC7686720

[17] van der Velde N Stricker BHC Pols HAP van der Cammen TJM. Risk of falls after withdrawal of fall-risk-increasing drugs: a prospective cohort study. Br J Clin Pharmacol. (2007) 63:232–7. doi: 10.1111/j.1365-2125.2006.02736.x, 16939531 PMC2000574

[18] Goto NA van Heel DAM Dautzenberg L Sibille FX Jennings E Bauer DC . Impact of discontinuation of fall-risk-increasing drugs on falls in multimorbid older patients with polypharmacy. J Am Geriatr Soc. (2025) 73:1827–35. doi: 10.1111/jgs.19460, 40167043 PMC12205291

[19] Blalock SJ Casteel C Roth MT Ferreri S Demby KB Shankar V. Impact of enhanced pharmacologic care on the prevention of falls: a randomized controlled trial. Am J Geriatr Pharmacother. (2010) 8:428–40. doi: 10.1016/j.amjopharm.2010.09.002, 21335296

[20] Ayhan Oncu Y Seren Intepeler S. Nurses’ view of implementation evidence-based fall prevention interventions: a qualitative study. J Nurs Manag. (2022) 30:234–42. doi: 10.1111/jonm.13480, 34591345

[21] Innab AM. Nurses’ perceptions of fall risk factors and fall prevention strategies in acute care settings in Saudi arabia. Nurs Open. (2022) 9:1362–9. doi: 10.1002/nop2.1182, 35099122 PMC8859041

[22] Wen J Wen J Jing W He H Ren J. The knowledge, attitude, and practice toward fall prevention of parturients in the obstetric inpatient wards: a comparative cross-sectional study between nurses and parturients. Front Public Health. (2025) 13:1412111. doi: 10.3389/fpubh.2025.1412111, 39949554 PMC11823421

[23] Munshi R Maurya M. Impact of educational intervention on the knowledge, attitude, and practice of pharmacovigilance among nurses at a tertiary care public hospital. Curr Drug Saf. (2023) 18:31–8. doi: 10.2174/1574886317666220426092537, 35473524

[24] Bowden V Bradas C McNett M. Impact of level of nurse experience on falls in medical surgical units. J Nurs Manag. (2019) 27:833–9. doi: 10.1111/jonm.12742, 30565782

[25] Lindberg DS Prosperi M Bjarnadottir RI Thomas J Crane M Chen Z . Identification of important factors in an inpatient fall risk prediction model to improve the quality of care using EHR and electronic administrative data: a machine-learning approach. Int J Med Inform. (2020) 143:104272. doi: 10.1016/j.ijmedinf.2020.104272, 32980667 PMC8562928

[26] Shafqat N Rawat T Singh V Verma R Rani M Tailor SK. Enhancing pediatric patient safety by evaluating the effect of targeted education on nurses’ knowledge, attitudes, and practices of fall prevention. Cureus. (2025) 17:e82946. doi: 10.7759/cureus.82946, 40416284 PMC12103634

[27] Gray-Miceli D de Cordova PB Crane GL Quigley P Ratcliffe SJ. Nursing home registered nurses’ and licensed practical nurses’ knowledge of causes of falls. J Nurs Care Qual. (2016) 31:153–60. doi: 10.1097/NCQ.0000000000000157, 26421775

[28] Negash NA. Assessment of self-reported practice of nurses towards fall prevention and its associated factors in an Ethiopian hospital; cross-sectional study. Int J Orthop Trauma Nurs. (2022) 46:100960. doi: 10.1016/j.ijotn.2022.100960, 35917679

[29] Wake AD Tuji TS Gonfa BK Waldekidan ET Beshaw ED Mohamed MA . Knowledge, attitude, practice and associated factors towards patient safety among nurses working at Asella referral and teaching hospital, Ethiopia: a cross-sectional study. PLoS One. (2021) 16:e0254122. doi: 10.1371/journal.pone.0254122, 34197548 PMC8248719

